# Immunogenic Cell Death and Immunotherapy of Multiple Myeloma

**DOI:** 10.3389/fcell.2019.00050

**Published:** 2019-04-16

**Authors:** Alfonso Serrano-del Valle, Alberto Anel, Javier Naval, Isabel Marzo

**Affiliations:** Department of Biochemistry and Molecular and Cell Biology, University of Zaragoza, Zaragoza, Spain

**Keywords:** immunogenic cell death, multiple myeloma, ER stress, danger-associated molecular pattern, immunotherapy

## Abstract

Over the past decades, immunotherapy has demonstrated a prominent clinical efficacy in a wide variety of human tumors. For many years, apoptosis has been considered a non-immunogenic or tolerogenic process whereas necrosis or necroptosis has long been acknowledged to play a key role in inflammation and immune-related processes. However, the new concept of “immunogenic cell death” (ICD) has challenged this traditional view and has granted apoptosis with immunogenic abilities. This paradigm shift offers clear implications in designing novel anti-cancer therapeutic approaches. To date, several screening studies have been carried out to discover *bona fide* ICD inducers and reveal the inherent capacity of a wide variety of drugs to induce cell death-associated exposure of danger signals and to bring about *in vivo* anti-cancer immune responses. Recent shreds of evidence place ER stress at the core of all the scenarios where ICD occur. Furthermore, ER stress and the unfolded protein response (UPR) have emerged as important targets in different human cancers. Notably, in multiple myeloma (MM), a lethal plasma cell disorder, the elevated production of immunoglobulins leaves these cells heavily reliant on the survival arm of the UPR. For that reason, drugs that disrupt ER homeostasis and engage ER stress-associated cell death, such as proteasome inhibitors, which are currently used for the treatment of MM, as well as novel ER stressors are intended to be promising therapeutic agents in MM. This not only holds true for their capacity to induce cell death, but also to their potential ability to activate the immunogenic arm of the ER stress response, with the ensuing exposure of danger signals. We provide here an overview of the up-to-date knowledge regarding the cell death mechanisms involved in situations of ER stress with a special focus on the connections with the drug-induced ER stress pathways that evoke ICD. We will also discuss how this could assist in optimizing and developing better immunotherapeutic approaches, especially in MM treatment.

## Introduction

Every day in the human body, billions of cells pass away and are kindly replaced by newborn members leaving no trace behind, allowing in this way conservation of whole-body homeostasis. In order to occur without catastrophic consequences, this process must remain almost completely unnoticed to the immune system. During this physiological, programmed cell death, mainly in the form of apoptosis, intracellular content is confined within membranous bodies that are rapidly cleared by phagocytes in an immunological “silent manner” Hence, apoptosis has long been considered a non-immunogenic or even tolerogenic process, whereas necrosis and necroptosis have been shown to play a key role in inflammation and immune related processes (Poon et al., 2014; Yatim et al., 2015). However, the new concept of “immunogenic cell death” (ICD) has challenged this traditional view and has granted apoptosis with immunogenic abilities. This immunostimulatory kind of apoptosis is characterized by the ability of dying cells to elicit robust adaptive immune responses against altered self-antigens/cancer-derived neo-epitopes, in the case of tumor cells, or against pathogen-derived antigens (Ags) during the course of an infection (Galluzzi et al., 2017). Besides antigenicity, another vital factor needed to unleash a genuine immune response is adjuvanticity, which is conferred by microorganism- and/or danger-associated molecular patterns (MAMPs and DAMPs, respectively). These are molecules that are exposed or released by dying cells and let the immune system know the existence of a menace to the organism (Fuchs and Steller, 2015). This “danger” state is sensed in the human body by pattern recognition receptors (PRRs) displayed by innate immune cells such as monocytes, macrophages and dendritic cells (DCs), hence promoting activation and maturation of these cells to engage the adaptive arm of the immune system (Matzinger, 2002).

Screening studies have been carried out to unveil the immunogenic potential of myriads of anti-cancer agents (Sukkurwala et al., 2014). To date, only a small yet diverse collection of anti-cancer therapies, whether chemotherapeutic drugs (e.g., anthracyclines, oxaliplatin, bortezomib) (Obeid et al., 2007; Garg et al., 2017) or physical modalities [e.g., radiotherapy, hypericin-based photodynamic therapy (Hyp-PDT), and high hydrostatic pressure (HHP)] (Golden et al., 2012; Adkins et al., 2014) have been shown to induce bona-fide ICD. However, a common denominator can be extracted from the action mechanisms of all these approaches: ER stress and ROS generation. Thus, activation of the ER stress pathways also known as the unfolded protein response (UPR), and specially, the PERK-mediated arm of the UPR is vital for the vast majority, if not all, the scenarios where ICD occurs (Rufo et al., 2017). Moreover, during tumor development, cancer cells have to cope with harsh conditions that trigger ER stress. Thus, UPR activation constitutes an important hallmark of several human cancers that endow cancer cells with the ability to acquire essential characteristics required for tumor progression (Corazzari et al., 2017). Of note, although UPR activation is initially intended to restore cell homeostasis, it can also shift the cellular fate toward cell death. All the aforementioned has clear implications for cancer therapy. The UPR-dependency of tumor cells together with the connection of ER-stress and the emission of danger signals (or ER stress-ICD connection), can be harnessed to design novel therapeutic tools. These therapeutic approaches not only would reduce tumor burden, but also improve the immunogenic capacity of dying cancer cells to elicit long-term adaptive immune responses. In particular, in multiple myeloma (MM), a lethal plasma cell disorder, the elevated production of immunoglobulins leaves these cells heavily reliant on the survival arm of the UPR. Nevertheless, although myeloma cells rely on the UPR to thrive, they are extremely sensitive to ER-stress associated cell death. This feature explains why proteasome inhibitors show a prominent clinical efficacy in the treatment of MM (Merin and Kelly, 2014; Scalzulli et al., 2018). Sadly, resistance to therapy is recurrent, and in most of the cases accounts for the lethality of the disease (Robak et al., 2018). MM is also a genuine neoplasia where the immune system is compromised. Nonetheless, immunotherapeutic interventions in this disease have potential to be successful, as graft-vs-myeloma effect has been evidenced in patients subjected to allogenic stem-cell transplantation or under donor lymphocyte infusions (Ladetto et al., 2016). In fact, current immunotherapeutic approaches are giving promising results in relapsed and refractory patients. Among the novel and more promising immune-based therapies that are under investigation, we can include: (1) Antibody-based therapies with daratumumab and elotuzumab as the flagships of this kind of approach, (2) Boost the immune effector line of defense with adoptive cell therapy (ACT), either with expanded tumor-infiltrating lymphocytes (TILs), NK cells or CAR-T cells, (3) Releasing the brakes of immune response with immune-checkpoint blockade, (4) Enhancing general anti-tumor immunity through vaccination strategies, and finally (5) Combinatorial strategies of the immunotherapies themselves or combined with immunogenic chemo- or radiotherapies. Noteworthy, all of these approaches can theoretically be benefited by ICD. Hence, the immunostimulatory potential of chemotherapeutics or other ICD-related modalities could be exploited to enhance general immunity or at least create an immune-friendly tumor microenvironment. This way, some of the drawbacks occurring in the clinical setting could be circumvented to achieve an effective immune response in cancer patients (Montico et al., 2018).

## The Unfolded Protein Response

Tumor cells are constantly coping with aggressive insults and subjected to different types of cellular stress. Some of these extrinsic (hypoxia, nutrient deprivation, acidosis) and intrinsic (oncogenic activation, genetic alterations, exacerbated secretory capacity) factors are common instigators of ER stress (Dufey et al., 2016). To cope with ER stress, cells activate an adaptive and well-conserved mechanism called UPR. The UPR is a fine-tuned process controlled by three membrane-bound ER stress sensors: Protein Kinase RNA-activated (PKR)-like ER Kinase (PERK), Inositol-Requiring transmembrane kinase/Endonuclease (IRE1) and Activating Transcription Factor 6 (ATF6). These sensors remain inactive in basal conditions due to the interaction with Binding Immunoglobulin Protein (BIP, also known as GRP78) through their ER luminal domains. Under ER stress conditions, BIP dissociates from the ER stress sensors to help in protein folding (Almanza et al., 2018). This event allows ER stress sensors to self-activate by homodimerization/oligomerization and trans-auto-phosphorylation in the case of PERK and IRE1, and translocation to the Golgi in the case of ATF6. First, the UPR tries to restore cell homeostasis, by attenuating protein translation, enhancing degradation of misfolded proteins and increasing levels of ER chaperones and redox enzymes to increase folding capacity (Almanza et al., 2018). However, if ER stress persists, the UPR can trigger proapoptotic programs controlled mainly by the IRE1 and PERK arms. IRE1 is a Ser/Thr kinase that also has an endoribonuclease domain. When activated, IRE1 drives XBP1 mRNA splicing, leading to a more stable XBP1s protein that acts as a transcription factor upregulating genes controlling ER homeostasis maintenance (Sano and Reed, 2013). Moreover, during the chronic phase of ER stress, IRE1 is also able to degrade many ER-targeted mRNAs through regulated IRE1-dependent mRNA decay (RIDD) process. Activation of PERK signaling leads to phosphorylation of eIF2α, which results in inhibition of global protein translation in order to reduce protein load. Nonetheless, some transcripts like ATF4 are translated more efficiently during ER stress. ATF4 increases the expression of genes involved in aminoacid and redox metabolism, ubiquitin ligases and the transcription factor CAAT/enhancer-binding protein (C/EBP) homologous protein (CHOP/GADD153). ATF4 and CHOP are also key determinants of ER stress-induced cell death. Finally, the cytosolic domain of ATF6 also acts as a transcription factor that mainly regulates the expression of genes involved in the ER-associated degradation (ERAD) pathway (Dufey et al., 2016).

## Immunogenic Cell Death

During the last decade, our conception of the characteristics of different types of cell death has significantly changed. Necrosis was first conceived as an accidental, pathological and pro-inflammatory form of cell death, whereas apoptosis was recognized to be a non-immunogenic, physiological and regulated way of cell demise (Poon et al., 2014). However, these features are no longer so clear-cut since programmed necrosis (necroptosis) has been shown to be triggered by a genetically encoded, well-regulated molecular program (Golstein and Kroemer, 2007; Vanden Berghe et al., 2009; Dhuriya and Sharma, 2018). On the other hand, apoptosis is no longer considered to be an immunologically “silent” process, since some apoptotic cells are able to induce antigen-specific immune responses (Obeid et al., 2007). In cancer research, the role of the immune system has been overlooked for many years due, in part, to the way chemotherapy and other anticancer therapies were usually tested. Particularly, the frequent use of immunodeficient mice to assess the efficacy of these treatments has precluded from gaining insight on the precise role of the immune system in cancer therapy (Krysko et al., 2012). Nonetheless, the re-evaluation of concepts like cancer antigenicity and ICD, as well as the interpretations from Danger Theory, has redirected the focus in oncological research toward novel or improved immunotherapeutic protocols (Garg et al., 2015a).

**FIGURE 1:**
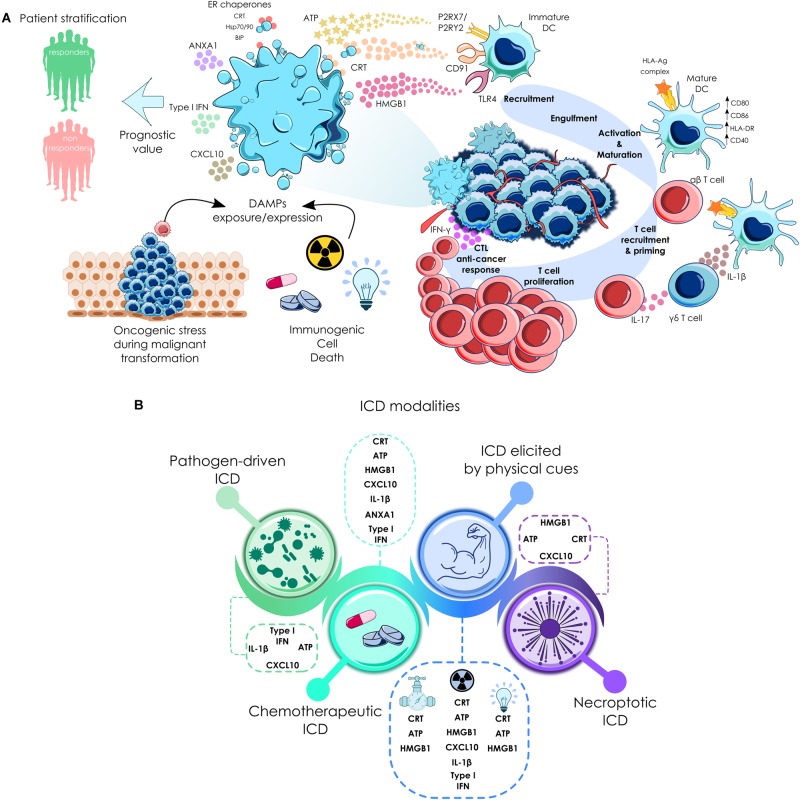
Immunogenic cell death cycle and forms of ICD. **(A)** Cancer cells subjected to some type of chemotherapeutics and other anti-cancer therapies expose calreticulin (CRT) and other endoplasmic reticulum chaperones, such as Hsp70, Hsp90 or Bip on their surface, secrete ATP, initiate type I interferon (IFN) response that is able to trigger the production of CXC-chemokineligand 10 (CXCL10), and release high-mobility group box 1 (HMGB1) and annexin A1 (ANXA1). When secreted or exposed extracellularly, they bind to their cognate receptors on the surface of myeloid or lymphoid cells, which enables the engulfment of cell corpses by antigen-presenting cells, including DCs. This process in the context of proper immunostimulatory signals, eventually leads to the priming of an adaptive immune response involving both αβ and γδ T cells. This culminates in the establishment of a CTL-mediated anti-cancer immune response with potential to kill therapy-resistant cancer cells via an IFNγ-dependent mechanism. In the clinical setting, cancer cells with higher expression of some DAMPs have been found. Depending on the cancer type, this could be correlated with good or bad prognosis, as well as to markers of an active anti-cancer immune response. **(B)** Forms of ICD. Different variants of ICD could be evoked by distinct types of stimuli that are associated with a differential set of danger signals. Even in the form of immunogenic chemotherapy, each drug could instigate differential danger signaling pathways (Galluzzi et al., 2017); P2RX7, purinergic receptor P2X7; P2RY2, purinergic receptor P2Y2; TLR4, Toll-like receptor 4.

The ICD concept has been defined as an unique class of regulated cell death capable of eliciting complete antigen-specific adaptive immune responses through the emission of a spatiotemporally defined set of danger signals or DAMPs (Casares et al., 2005; Kroemer et al., 2013). These signals are endogenous molecules that perform conventional intracellular functions but when extracellularly exposed, gain immunogenic competences. The release or membrane exposure of these molecules, allow their interaction with their cognate PRRs displayed by innate immune cells such as monocytes, macrophages and DCs. This leads to activation and maturation of these cells that migrate to draining lymph nodes loaded with cancer-derived antigen-specific cargoes. Cancer antigens are then presented to T cells (CD4^+^ and CD8^+^ T lymphocytes) which enable a potent anticancer adaptive immune response (Chen and Mellman, 2013). To date, four modes of ICD have been described, each related to a particular type of inducing stimulus and to the emission of a specific set of danger signals (Galluzzi et al., 2017) (see Figure 1): (1) Pathogen-driven ICD, as one of the defense mechanisms against invading pathogens; (2) ICD exhibited by physical cues, such as Hyp-PDT, irradiation and HHP; (3) Necroptosis, but not accidental necrosis, since this regulated form of cell death was able to vaccinate syngenic mice against a rechallenge with cells of the same type (Aaes et al., 2016). According to this, RIPK3 or MLKL deficiency abrogated the ability of these cells to secrete the required immunogenic signals that lead to an anticancer immune response in mice (Yang et al., 2016); and (4) ICD evoked by some chemotherapeutics targeting different types of essential cell components or processes that induce cell death pathways. It has been demonstrated that a diverse panel of drugs can elicit protective immune responses in mice (Apetoh et al., 2007; Obeid et al., 2007; Michaud et al., 2011). Of note, despite some screening studies using large drug libraries have been performed, only a small group of candidates have emerged to be valid ICD inducers (Obeid et al., 2007; Martins et al., 2011; Menger et al., 2012; Sukkurwala et al., 2014). The chemical nature of these agents, is considerably diverse: oxazophorines like cyclophosphamide (Schiavoni et al., 2011); Pt-based compounds as oxaliplatin (Tesniere et al., 2009); anthracyclines (Minotti et al., 2004) such as idarubicin and doxorubicin; anthracenediones such as mitoxantrone and dipeptides such as bortezomib (Merin and Kelly, 2014). Similar to bortezomib, carfilzomib another proteasome inhibitor used in the treatment of MM, has also shown to expose CRT in different MM cell lines (Jarauta et al., 2016). Although it may appear attractive, no simple structure-function relationship has been found that could predict the suitability of drugs to trigger ICD. This is clearly exemplified by the oxaliplatin-cisplatin or the melphalan-cyclophosphamide paradigms (Tesniere et al., 2009; Martins et al., 2011; Dudek-Perić et al., 2015). Several factors, such as the type of cell death, the ICD stimuli and the interconnection between various cellular stress responses, influence the type of danger signals emitted during the course of cell death (Agostinis, 2017). On the other hand, combinatorial strategies can be exploited to compensate for DAMPs generation scarcity displayed by some agents, restore immunogenicity and hence transform tolerogenic cell death into immunogenic modalities (Martins et al., 2011; Bezu et al., 2015). Furthermore, not all the DAMPs exposed during cell death are immunostimulatory. In fact, there are some molecules (Prostaglandin E2, adenosine, etc.) (Agostinis, 2017; Galluzzi et al., 2017) that exhibit immunosuppressive properties and play important roles in tolerance to dead cells. Among all members of DAMP family, the best studied, and those who have been shown to be pivotal for ICD are described in the next section.

### Calreticulin and ER Chaperones

Calreticulin (CRT) is a, highly conserved, soluble, ER-associated chaperone with numerous functions inside and outside the ER (calcium homeostasis, assembly of MHC-I, etc.) (Johnson et al., 2001; Michalak et al., 2009). In stressed or dying cells, CRT is exposed in the outer leaflet of the plasma membrane (ecto-CRT) where it functions as a potent “eat-me” signal. CRT binds to LRP1 (also known as CD91), and possibly other scavenger receptors, displayed by phagocytic cells. This role in phagocytic clearance of dead cells was first described by Gardai et al. (2005). Nonetheless, Obeid et al. (2007) went a step further and demonstrated that CRT exposure was a key determinant in ICD-driven anticancer immunity. Actually, cancer cells undergoing cell death triggered by certain chemotherapeutics, expose CRT on their surface. This event leads to the engulfment of cancer material by DCs and, more importantly, to tumor antigen presentation and anticancer cytotoxic T lymphocyte (CTL) specific responses (Kroemer et al., 2013). Furthermore, ecto-CRT has been shown to prompt IL-6 and TNF expression on DCs, priming pro-inflammatory T-helper type 17 (Th17) polarization (Pawaria and Binder, 2011). Likewise, other ER-resident chaperones such as heat-shock protein 70 (Hsp70) and Hsp90, play also an important role in the immunogenicity of dying cancer cells. Thus, ecto-Hsp90 has been reported to enhance DC uptake of bortezomib-treated MM cells, including primary cells isolated from MM patients and induction of anticancer immunity (Spisek et al., 2007). On the contrary, Dudek-Perić et al. (2015), using blocking antibodies against Hsp90 in a DC maturation assay, reported that this chaperone was not (or at least partially) involved in the immunogenicity of melanoma cells treated with melphalan. The specific role of Hsp70 in the immunogenicity of cancer cells has not been studied so extensively. However, it has been reported that in shikonin- or gemcitabine-treated cells, Hsp70 was involved in DC-mediated activation of CD4^+^ and CD8^+^ T cells (Pei et al., 2014; Lin et al., 2015). In the case of Hyp-PDT treatment, Hsp70 promotes nitric oxide (NO) generation in innate immune cells (Song et al., 2013). In a different context, Hsp70 has shown to efficiently vaccinate mice against murine MM cells using a DNA-based vaccination strategy (Liu et al., 2018). BIP, a fundamental regulator of ER function and the UPR, has been described to be secreted and participate in the cross-presentation of tumor-derived Ags in DCs, inducing Ag-specific CTL immune responses (Tamura et al., 2011). Indeed, chaperones as efficient protein folding mediators, are often present bound to antigenic peptides. When released, these chaperone-peptides complexes enter APCs by endocytosis via CD91 receptors and are cross-presented on MHC-I and MHC-II molecules to CD8^+^ and CD4^+^ T cells (Feng et al., 2001, 2003). Thereby, these molecules not only potentiate immunogenicity of dying cancer cells by acting merely as potent danger signals, but also contribute to boost cancer antigenicity assisting in the cross-presentation process.

**FIGURE 2:**
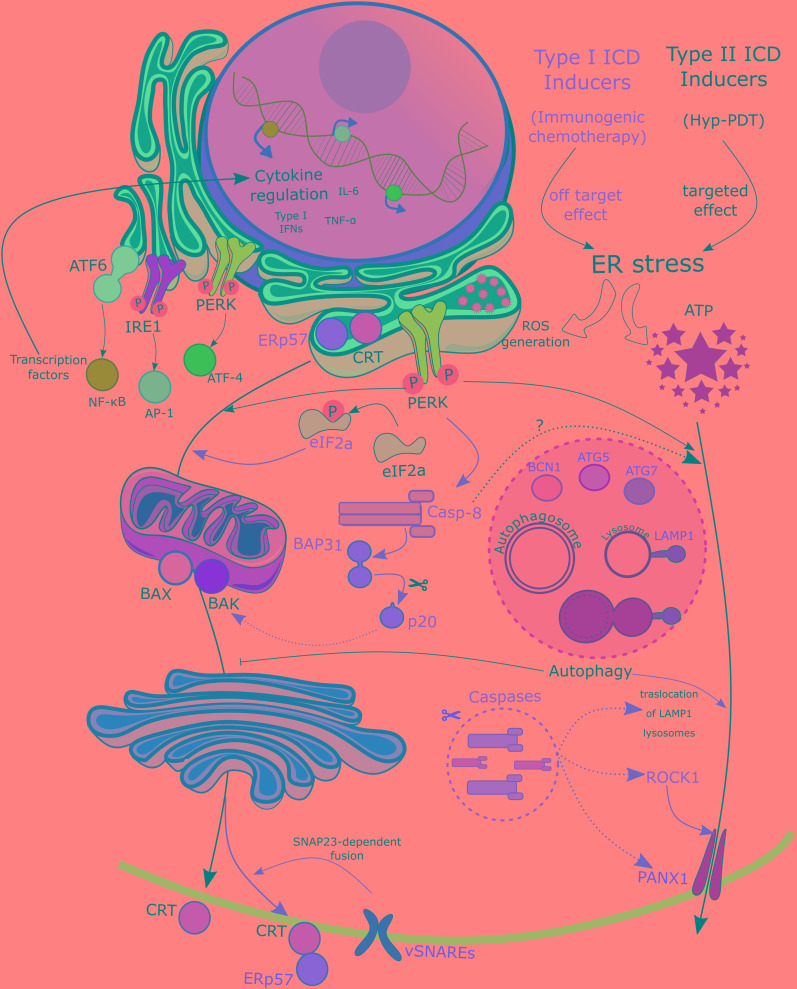
Mechanisms of DAMPs exposure. Differential mobilization pathways can be observed between Type I and Type II ICD inducers, defined by their off-targeted or targeted effect on the ER, respectively. Exposure of CRT in the plasma membrane upon treatment with Type I ICD inducers requires an intrincate pathway with activation of the ER stress–ROS signaling mediated by the activation of the PERK, and the ensuing phosphorylation of eiF2α. This is followed by the required cleavage of B-cell receptor-associated protein 31 (BAP31) by preapoptotic caspase-8. Bax/Bak activation is also mandatory in this process. Finally CRT relocation also requires anterograde ER-Golgi trafficking and the exocytic pathway in a SNAP23-dependent manner (Panaretakis et al., 2009). Along all the way from the ER to the plasma membrane, CRT is accompanied by ERp57. Upon treatment with Type II ICD inducers fewer requirements are needed, since this pathway only relies on PERK, Bax, Bak, and the secretory pathway. Regarding ATP secretion upon type II ICD inducers treatment, it follows a pathway quite similar to that of CRT, except for Bax/Bak and involving partially caspase 8. Type I ICD inducers require an independent pathway mediated by autophagy as ATG5, ATG7 and BCN1 are required in ATP release. Moreover, other molecules involved in different cellular processes like lysosomal exocytosis (LAMP1), membrane blebbing (ROCK1), apoptotic machinery (caspases) and membrane permeabilization (PANX1) have been shown to be essential in type I ICD-induced ATP externalization (Martins et al., 2014). CRT, calreticulin; eIF2a, eukaryotic initiation factor 2; ER, endoplasmic reticulum; ICD, immunogenic cancer cell death; PANX1, Pannexin 1; PERK, protein kinase R-like ER kinase; PI3K, phosphatidylinositol-4,5-bisphosphate 3-kinase; ROCK1, rho-associated, coiled-coil-containing protein kinase 1; ROS, reactive oxygen species; SNARE, SNAP (soluble N-ethylmaleimide-sensitive factor attachment protein) receptor.

With regards to the kinetics and the cellular pathways involved in the exposure of CRT (depicted in Figure 2), it has been documented that they may differ depending on both the apoptotic phase under evaluation and the inducing stimulus (Krysko et al., 2012). For example, there are some instances where ecto-CRT exposure precedes phosphatidylserine externalization (Panaretakis et al., 2009; Osman et al., 2017), is systemically accompanied by ERp57 to the plasma membrane and requires PERK-mediated phosphorylation of eIF2α. This is followed by caspase-8 activation and specific cleavage of BAP31, leading to the subsequent activation of BAX and BAK. CRT relocation also requires anterograde ER-Golgi trafficking and the exocytic pathway in a SNAP23-dependent manner (Panaretakis et al., 2009). On the contrary, Hyp-PDT mediated CRT exposure requires PERK, BAX, BAK and the secretory pathway but not eIF2α phosphorylation and caspase-8 activation (Garg et al., 2012c). However, there are other ways by which CRT can be relocated to the cell surface and that are independent from the aforementioned mechanisms. Other studies claimed that CRT can bind with high-affinity to phosphatydilserine (Païdassi et al., 2011; Wijeyesakere et al., 2016) in a Ca^2+^-dependent manner, and thus during cell death these two molecules are co-translocated at the same time in a caspase-independent fashion (Tarr et al., 2010).

Many studies investigating the role of CRT in ICD, carried out either *in vitro* or using *in vivo* animal models, assume the fact that CRT exposure is a consequence of the therapy itself. However, these studies have not considered basal surface expression of CRT on cancer cells and its potential implication on immunogenicity. Clinical studies supporting tumor cell-dependent immunity associated to basal CRT exposure are scarce and direct immunogenic effects of cells killed by chemotherapy in cancer patients have been rarely observed. It has been proposed that this is probably due to the fact that the chemotherapeutic dose needed to efficiently induce ICD is not reached in the clinical practice (Montico et al., 2018). Most of the available data indicate that tumor tissues express higher levels of CRT than healthy tissues, and that CRT expression may correlate with cancer progression and aggressiveness (Fucikova et al., 2018). Moreover, increasing clinical evidence is supporting the notion that CRT exposure, as well as other DAMPs may serve as important prognostic biomarkers in cancer patients (Fucikova et al., 2018). Different studies have shown that, depending on the cancer cell type, CRT expression could stand as a positive or negative prognostic factor for cancer patients. For example, in acute myeloid leukemia (AML), indolent B-cell lymphoma, non-small cell lung cancer (NSCLC), ovarian cancer, glioblastoma, endometrial cancer or colon cancer, the increased expression of CRT correlates with a favorable clinical outcome, as well as (in some cases) with increased levels of biological markers related to an active anti-cancer immune response (Peng et al., 2010; Zappasodi et al., 2010; Garg et al., 2015b; Stoll et al., 2016; Fucikova et al., 2016a,b, 2018; Xu et al., 2018). Meanwhile, in other cancer types like gastric cancer, pancreatic cancer, neuroblastoma, bladder carcinoma and mantle cell lymphoma, higher CRT levels were related to a poor clinical outcome (Chen et al., 2009; Chao et al., 2010; Sheng et al., 2014). In some cases like in esophageal squamous carcinoma, no differences in overall survival between CRT-high and low expression groups were found (Suzuki et al., 2012; Fucikova et al., 2018). In some of these studies, other markers involved in ICD or ER stress response such as phosphorylation of eIF2α, Hsp70, Hsp90 and BiP (GRP78/HSPA5), correlated with CRT expression and patient prognosis (Uramoto et al., 2005; He et al., 2011; Fucikova et al., 2016a,b). As mentioned above, only in a few studies a correlation between increased CRT expression and the chemotherapy regimen and good prognosis was found. For example, ovarian tumors from patients that displayed high levels of CRT showed a good clinical response to radiotherapy or treatment with paclitaxel (which are well-known ICD inducers) (Garg et al., 2015b). Similarly, in endometrial cancer patients, low CRT expression was associated with poor survival rates and resistance to doxorubicin (another reported ICD inducer) (Xu et al., 2018). However, in other cases such as in patients with NSCLC or AML, cancer cells exposed heterogeneous levels of CRT, regardless of the treatment received. Cancer cells can also experiment stress prior to chemotherapy, perhaps due to the oncogenic malignant transformation itself (Fucikova et al., 2018). This alternative source of stress also activates ER stress responses culminating in CRT translocation and danger signaling (Fucikova et al., 2018). This process facilitates anti-cancer immunosurveillance, represented by the higher amount of infiltrating mature DCs and effector T cells in the case of NSCLC patients (Stoll et al., 2016) and increased numbers of circulating NK cells and IFN-γ producing CD4^+^ and CD8^+^ T cells in AML patients (Fucikova et al., 2016b). Moreover, cancer cells that express low levels of CRT have shown to correlate, in some cases, with therapy resistance, such as in endometrial cancer patients (Xu et al., 2018). It is possible that this reduced CRT expression may arise from the ability of cancer cells to resist ER stress conditions (whether oncogenic- or chemotherapy-driven). Therefore, this situation might be overcome by using ER stressors that directly target ER stress response, possibly sensitizing to conventional chemotherapy and restoring danger signaling and the ensuing anti-cancer immunosurveillance.

### ATP

During the course of ICD, dying cells expel ATP (Ghiringhelli et al., 2009; Michaud et al., 2011) to the extracellular milieu where it functions as a powerful short-range “find me” signal (Elliott et al., 2009). Once secreted, ATP binds to ionotropic (P2X7) and metabotropic (P2Y2) purinergic receptors on APCs (Elliott et al., 2009; Ghiringhelli et al., 2009), stimulating their phenotypic maturation and chemotactic attraction, respectively (Galluzzi et al., 2015). In particular, extracellular ATP can activate the caspase-1 dependent NLRP3 complex (the so called inflammasome) triggering IL-1β secretion (Ghiringhelli et al., 2009), which in turn promotes CD8^+^ T cell (Ghiringhelli et al., 2009), as well as, IL-17 producing-γδ T cell (Ma et al., 2011) anti-tumor responses. According to this, mice lacking any of these components (Nlrp3^-/-^, P2rx7^-/-^ or Casp1^-/-^) seem to be incapable of promoting adaptive immune responses during drug-induced ICD (Ghiringhelli et al., 2009; Ma et al., 2011). The molecular mechanisms of ATP secretion during ICD are also dependent on ICD-inducing stimulus. In mitoxantrone- or oxaliplatin-driven early apoptotic ATP secretion, autophagy has been demonstrated to be mandatory, since depletion of important autophagy proteins (ATG5, ATG7 and BCN1) prevented ATP release (Martins et al., 2014). Moreover, other molecules involved in other cellular processes such as lysosomal exocytosis (LAMP1, VAMP1), membrane blebbing (ROCK1, myosin II), apoptotic machinery (caspases) and membrane permeabilization (pannexin 1, PANX1) have been shown to be essential for ICD-induced ATP release (Martins et al., 2014). Interestingly, PANX1 activation and surface exposure, as well as, LAMP1 translocation are strongly dependent on caspases rather than on the autophagic machinery (Martins et al., 2014). In fact, it is possible that remodeling of autophagic effectors and lysosomal effectors or PANX1 hemichannels by caspases rather than the mere presence of these components *per se*, are the real originators of ATP secretion (Garg et al., 2014; Martins et al., 2014). However, as it occurs in Hyp-PDT induced CRT relocation, ATP secretion mechanisms may differ from those described for chemotherapy-induced ICD. In particular, Hyp-PDT mediated ATP is autophagy independent (Garg et al., 2013) and rather requires the PERK-mediated proximal secretory pathway and PI3K-regulated exocytosis (Garg et al., 2012c).

### HMGB1

High mobility group Box 1 is a non-histone chromatin-binding protein localized in the nucleus, where it interacts with DNA and regulates transcription (Garg et al., 2010). In particular, it regulates the activity of NF-κB and p53 and other transcription factors and favors VD(J) recombination (Müller et al., 2004; Krysko et al., 2012). Extracellularly, HMGB1 can perform cytokine-based (distinct from DAMP-based) functions in monocytes and macrophages under the influence of pro-inflammatory molecules (TNF, LPS, IL-1β) (Scaffidi et al., 2002; Müller et al., 2004; Krysko et al., 2012). When released from dying cells, HMBG1 exerts potent immunostimulatory effects by interacting with distinct PRRs (TLR2, TLR4 and RAGE) (Sims et al., 2010). During chemotherapy- or radiotherapy-induced cell death, HMGB1 is released from dying cells and signals through TLR4-MyD88 axis on DCs, facilitating antigen processing and presentation (Apetoh et al., 2007; Saenz et al., 2014). The molecular pathways that participate in release of this DAMP remains to be elucidated. It has been documented that necrotic cells passively release huge amounts of HMGB1, acting as a potent mediator of inflammation (Scaffidi et al., 2002). Similarly, HMGB1 is also released by secondary necrotic cells and the use of Z-VAD-fmk (a broad caspase inhibitor that delays secondary necrosis) impede HMGB1 discharge in cells undergoing ICD (Bell et al., 2006; Apetoh et al., 2007). The immune related features of HMBG1 are strongly influenced by its redox status (Venereau et al., 2012; Yang et al., 2012), and this may account for the observed contradictory results (Palumbo et al., 2004; Jube et al., 2012). This redox modulation as well as the different behaviors observed in different studies have precluded from drawing definitive conclusions (Garg et al., 2014).

## ICD – ER Stress Connection

As stated before, numerous studies have been carried out to decipher ICD mechanisms and large screening studies (Martins et al., 2011; Menger et al., 2012; Sukkurwala et al., 2014) have been performed to unveil the immunogenic potential of myriads of anti-cancer agents. All this work has converged toward a common denominator in ICD molecular pathways: ER stress and ROS generation (Tesniere et al., 2008; Rufo et al., 2017). Then, activation of the ER stress control pathways, also known as the UPR, and specially the PERK-mediated arm, is vital for the vast majority if not all the scenarios where ICD occurs (Panaretakis et al., 2009; Rufo et al., 2017). As mentioned in previous sections, CRT exposure induced by chemotherapeutics requires ER stress with a decisive participation of PERK-mediated phosphorylation of eIF2α (Panaretakis et al., 2009). Meanwhile, in hypericin-PDT induced ICD, the ER stress module is similarly required being PERK fundamental, but not eIF2α phosphorylation. Here, PERK may modulate proper secretory pathway functioning, in both ecto-CRT induction and ATP secretion (Garg et al., 2012c; van Vliet et al., 2015). Regardless of these dissimilarities, PERK abrogation through genetic maneuvers, significantly diminished (but not completely abolished) the immunogenicity of stressed cancer cells *in vivo* (Panaretakis et al., 2009; Garg et al., 2012c). Altogether, PERK have shown to be a major player in ICD-derived emission of danger signal(s). Depending on the trigger stimuli it could be involved only in CRT emission or both in ATP and CRT emission (Kepp et al., 2013; van Vliet et al., 2015; Rufo et al., 2017). Nevertheless, this context dependency determines whether PERK contribution arise from its UPR-related function (Panaretakis et al., 2009) or through its ability to modulate the proximal secretory pathway (Garg et al., 2012c). Moreover, other novel PERK cellular functions related to actin cytoskeleton dynamics and formation of ER-plasma membrane contact sites, may sustain DAMP trafficking in ICD (van Vliet et al., 2015, 2017; van Vliet and Agostinis, 2016; Rufo et al., 2017). Interestingly, although the three branches of the UPR (PERK, IRE1α and ATF6) were triggered under cardiac glycoside treatment (Menger et al., 2012), abrogation of IRE1α and ATF6 pathways through genetic interventions did not alter CRT exposure in dying cells under the influence of different types of therapies (mitoxantrone, oxaliplatin, UVC irradiation) (Panaretakis et al., 2009). Furthermore, tunicamycin and thapsigargin, two potent chemical ER stressors, both of which induce strong UPR responses (Obeng et al., 2006; Almanza et al., 2018; Shen et al., 2018), have been shown to efficiently restore CRT relocation and/or *in vivo* immunogenicity of cis-platinum or mytomicin C (Martins et al., 2011), two reported non-ICD inducers. Of note, it seems that ER stress alone is not sufficient to trigger CRT translocation or *in vivo* immune responses (Kepp et al., 2013). In line with this, tunicamycin and thapsigargin have been shown to be ineffective (or at least less effective as other *bona fide* ICD inducers) in eliciting ICD (Martins et al., 2011; Kepp et al., 2013). In contrast, thapsigargin has reflected the opposite in some scenarios (Peters and Raghavan, 2011). The relative importance of ER stress (the process itself and also its kinetics and intensity) is underscored by the classification of ICD inducers. There are two main groups of ICD inducers, type I and type II (Krysko et al., 2012; Rufo et al., 2017), depending on cell death is either a consequence of a primary effect of ER stress or death is triggered through a different path and ER stress is merely a secondary effect of the therapeutic agent under consideration (Krysko et al., 2012). For example, some oncolytic viruses (Newcastle disease virus) (van Vloten et al., 2018; Ye et al., 2018), Pt(II) N-heterocyclic carbene complex (Wong et al., 2015) and hypericin-PDT (Garg et al., 2012c) fall within type II ICD inducer category as they selectively target the ER provoking intense ROS-based ER stress (Krysko et al., 2012; Rufo et al., 2017). Conversely, anthracyclines (type I ICD inducers) exert its cytotoxic effects primarily on the nucleus, where they are mainly localized (Minotti et al., 2004) and leave the ER stress as a secondary side-effect. Bortezomib is also considered a type I ICD inducer. Although bortezomib affects ER homeostasis generating a potent ER stress response (Obeng et al., 2006; Verfaillie et al., 2013; Gandolfi et al., 2017; Manasanch and Orlowski, 2017) and elevation cellular ROS levels (Lipchick et al., 2016), its direct cellular target is the inhibition of 26S proteasome (Gandolfi et al., 2017). Thus, as the cellular targets of these two types of ICD inducers are different, it is conceivable that the cellular responses triggered (particularly in the ER stress context) are different both in their kinetics and potency. Consequently, this has clear implications in the quality and amount of danger signals emitted. In fact, it has been shown that hypericin-PDT (a type II ICD inducer) has a superior capacity of emitting faster, a higher number and a broader spectrum of DAMPs, compared to type I ICD inducers (Garg et al., 2012a,b,c; Krysko et al., 2012; Rufo et al., 2017).

It’s important to mention that, in some regulated variants of cell demise, ROS-mediated ER stress may be dispensable for triggering ICD and the ensuing *in vivo* immune responses (Aaes et al., 2016; Rufo et al., 2017). Specially, different to hypericin-PDT based and anthracycline-induced ICD, the necroptotic variant occurred in absence of apparent/perceptible ER stress or PERK activation (Aaes et al., 2016). This reveals that there may be alternative mechanisms that may take part in the induction of danger signaling and further reinforce the idea that ICD induction may be stimulus and context-dependent.

ER stress could also instigate immunosuppressive effects in the tumor microenvironment. In particular, transmissible ER stress has been observed in myeloid cells incubated with tumor supernatants obtained under ER stress conditions (Mahadevan et al., 2011; Colegio et al., 2014; Parker et al., 2015; De Sanctis et al., 2016). Moreover, tumor cells can activate in a paracrine fashion the UPR in tumor-infiltrated myeloid cells (DCs, MDSCs) that adopt an immunosuppressive phenotype, showing an impaired antigen presenting capacity, secretion of pro-inflammatory cytokines (IL-6, TNFα, IL-23, ...) as well as other immune-restraining factors (Mahadevan et al., 2011, 2012). Supporting this notion, mice tumors exposed to thapsigargin displayed exacerbated tumor growth which correlated with the increased numbers and aggressive phenotype of MDSCs (Lee et al., 2014). To our knowledge, although transmissible ER stress has not been directly demonstrated in MM, this system share common players with MM pathogenesis (IL-6, MDSCs, alteration of DCs). Therefore, as MM cell suffer from ER stress, it is not rare to think that transmissible ER stress might contribute to the characteristic immunosuppressive BM microenvironment in MM patients. Collectively, all these data seem to point to the fact that low to moderate ER stress may contribute to create an immunosuppressive environment, whereas high-level ER stress, such as the one occurred in ICD, could bring about immunostimulatory responses (Cubillos-Ruiz et al., 2017).

Besides the contributions to ICD stated before, ER stress may further boost DAMP signaling abilities of stressed cancer cells through the induction of autophagy (Martins et al., 2014; van Vliet et al., 2015). It is known that upon UPR activation, autophagy is activated as a defense mechanism to promote cell survival (Høyer-Hansen and Jäättelä, 2007; Velasco et al., 2010; Michallet et al., 2011; Corazzari et al., 2017). Moreover, as mentioned in previous sections, autophagy plays a crucial role in ATP secretion during ICD driven by chemotherapeutics (Martins et al., 2014). For these reasons, it may seem feasible that ER stress-induced autophagy triggered by ICD inducers further contributes to the immunogenicity of dying cancer cells. However, whether autophagy is directly induced by these drugs or is just a consequence of ROS-based ER stress in the context of ICD, needs to be thoroughly explored. Nonetheless, there are at least three facts that question the involvement of ER stress-induced autophagy in ICD: (1) The extensive characterization of molecular pathways involved in autophagy-mediated ATP secretion comprise molecular mechanisms (caspases, LAMP1-dependent trafficking, PANX1 channels lysosomal exocytosis) that seem to be independent of ER stress/UPR pathways (Martins et al., 2014). (2) In chemotherapy-induced ICD, autophagy do not regulate the emission of DAMPs which are dependent on ER stress pathways (Panaretakis et al., 2009; Michaud et al., 2011; Martins et al., 2014). (3) Finally, ATP secretion and CRT exposure appear to follow a different time-course, since CRT mobilization has been shown to occur prior phosphatidylserine externalization, whereas ATP is expelled during the blebbing phase of apoptosis. Altogether, these considerations may point to ER stress and autophagy as two independent constituents of ICD, at least in chemotherapeutic-driven ICD. On the other side, under Hyp-PDT treatment, autophagy has also been shown to be activated and to confer resistance against ROS-mediated cytotoxicity of stressed cancer cells (Dewaele et al., 2011; Rubio et al., 2012). One might argue that as hypericin is a direct ER sensitizer (Garg et al., 2012a) (type II ICD inducer), autophagy is triggered as a consequence of ER stress induction. Meanwhile in type I ICD inducers, as ER stress is not the primary target, autophagy could be induced upon interaction with other cellular targets. Furthermore, the ICD pathways involved in danger signaling are not identical when triggered by type I or type II ICD inducers. Thus, contrary to chemotherapy-induced ATP secretion, in the Hyp-PDT scenario ATP secretion is not dependent on autophagy machinery (Garg et al., 2013). Outstandingly, autophagy was found to attenuate CRT translocation and DCs maturation as well as suppress DC-mediated proliferation of CD4 and CD8 T cells (Garg et al., 2013). This has been rationalized as the autophagy machinery is able to clear oxidized proteins and organelles (Rubio et al., 2012; Garg et al., 2013), which in turn would alleviate the ER retention system that becomes overwhelmed under ER stress conditions (Johnson et al., 2001; Wiersma et al., 2015). Hence, during Hyp-PDT treatment, ER stress and ROS production allow oxidized proteins to accumulate leaving the ER retention system saturated (Dewaele et al., 2011; Rubio et al., 2012). Under these conditions, autophagy inhibition would increase the amount of oxidized proteins (possibly by augmenting ROS-based ER stress) and would favor that ER resident chaperones such as CRT could escape from ER confinement (Johnson et al., 2001; Peters and Raghavan, 2011; Garg et al., 2013). Similarly, in a model of melanoma, in wild-type as well as in BRAF-resistant cells, concurrent silencing of ATG5 and treatment with a MEK-inhibitor (U0126), amplified the levels of ecto-CRT and ecto-HSP90 compared to those cells in which autophagy was intact (Martin et al., 2015). Additionally, emerging mechanisms underpinning the crosstalk between the autophagic flux and the endosomal pathway could contribute to unravel the interplay of autophagy in modulation of ER-stress driven DAMP trafficking (Kim et al., 2012; Hyttinen et al., 2013; McKnight et al., 2014; van Vliet et al., 2015). ER stress could also have an impact over intracellular ATP levels through stimulation of mitochondrial respiration and bioenergetics (Bravo et al., 2012). This way the cell fill their bioenergetic stores to restore cell homeostasis. Given the chemotactic power of ATP, by increasing its cellular levels, the cell may also be preparing to alert the immune system that something is wrong. Finally, ER stress and the UPR could also impact on cytokine production in multiple levels (PRRs, transcription factors involved in cytokine production, etc.) (Smith et al., 2018). The mechanisms involved in this process are out of the scope of this manuscript and have been recently reviewed in Reverendo et al. (2019) and Smith et al. (2018).

## ER Stress-Associated Cell Death

With all these players around the table, it seems tempting to target PERK and/or ER stress in cancer. In fact, ER stress as a target, is increasingly getting more adepts in the cancer crusade. During tumor development cancer cells have to cope with harsh conditions that are widely known to trigger ER stress (e.g., nutrient deprivation, hypoxia, acidic pH) (Sano and Reed, 2013). Thus, UPR activation constitutes an important hallmark of numerous human cancers (Riha et al., 2017). This process endows cancer cells with the ability to acquire essential characteristics (dormancy, resistance to therapy, tumor-driven angiogenesis, etc.) required for tumor progression (Sano and Reed, 2013; Corazzari et al., 2017; Mohamed et al., 2017). As stated before, ER stress could also negatively influence immunity at different levels, favoring this way tumor development (Mahadevan et al., 2011; De Sanctis et al., 2016; Cubillos-Ruiz et al., 2017). In the particular case of MM, their exacerbated secretory phenotype leave these cells heavily reliant on the survival arm of the UPR. Therefore, as plasma cell development and survival strongly relies on an intact UPR (Reimold et al., 2001; Iwakoshi et al., 2003), it does not seem unusual that UPR activity increases with MM progression (Nakamura et al., 2006). Furthermore, whole genome sequencing studies have revealed that MM patients frequently harbor mutations in genes related to the UPR (Chapman et al., 2011). Among the UPR mediators, XBP1 has been found to be overexpressed in MM and has also been identified to be mutated in a small subpopulation of patients (Carrasco et al., 2007; Bagratuni et al., 2010; Chapman et al., 2011; Nikesitch et al., 2018). Nevertheless, although myeloma cells count on the UPR to thrive, they are extremely sensitive to ER stress-associated cell death (Obeng et al., 2006; Ling et al., 2012; Gandolfi et al., 2017). This feature explains why proteasome inhibitors, have shown a prominent clinical efficacy in the treatment of MM (Leleu et al., 2018; Scalzulli et al., 2018), although resistance to therapy is recurrent and in most of the cases accounts for the lethality of the disease (Nikesitch et al., 2018; Robak et al., 2018). For these reasons, novel ER stress/UPR-targeting therapies have emerged. Given its important role in myeloma pathogenesis, novel drugs targeting the RNAse domain of IRE1 (4μ8C, MKC-3946, STF083010) have been developed. These drugs showed significant tumor growth inhibition in mouse myeloma models (Papandreou et al., 2011; Mimura et al., 2012), as well as in primary myeloma plasma cells (Papandreou et al., 2011). In addition, new potent and selective first-in-class inhibitors have been developed against PERK (GSK2606414 and the derived form GSK2656157) (Atkins et al., 2013; Hoi et al., 2013). These drugs have shown promising pre-clinical results in a model of pancreatic cancer (Atkins et al., 2013; van Vliet et al., 2015). Nonetheless, given the dual role of ER stress and UPR related pathways in cancer, a word of caution about needs to be taken when targeting these cellular pathways. On one side we may be inhibiting the pro-tumorigenic role of UPR mediators but in the other, we may reduce the immunogenicity of cancer cells dampening danger signaling (or vice versa). Therefore, future investigations assessing the repercussion on overall immunity, as well as cell-autonomous responses on cancer cells, on immunocompetent mice models are needed in order to truly evaluate the therapeutic relevance of these approaches in cancer.

Although UPR activation is initially conceived to restore cell homeostasis, it is also able to shift the cellular demise toward cell death. When ER stress persists, the UPR is able to trigger proapoptotic programs controlled mainly by IRE1 and PERK arms. Activated IRE1 can act as a docking platform to recruit other proteins such as the adaptor protein TRAF2, that subsequently tethers ASK1 which causes activation of JNK/p38 MAPK pathway. These downstream stress kinases, are reported to promote apoptosis in several ways. For example, JNK phosphorylation has been shown to inhibit the anti-apoptotic members Bcl-2, Bcl-xL and Mcl-1, while activating pro-apoptotic members BID and BIM (Deng et al., 2001; Lei and Davis, 2003; Almanza et al., 2018). As regards to p38 MAPK, it phosphorylates and activates transcription factor CHOP which contributes to apoptosis controlling several Bcl-2 family members (Yamaguchi and Wang, 2004; Puthalakath et al., 2007). As in the case of PERK signaling, it increases the expression of ATF4 and CHOP, two key determinants of ER stress-induced cell death. CHOP can increase the transcription of BH3-only proteins BIM (Puthalakath et al., 2007) and PUMA (Cazanave et al., 2010). Moreover, Noxa has been reported to be upregulated by ATF4 (Armstrong et al., 2010). ATF4/CHOP pathway also downregulates the expression of Bcl-2 and Mcl-1 anti-apoptotic proteins, contributing in this way to cell death (Puthalakath et al., 2007; Gomez-Bougie et al., 2016). Moreover, PUMA, BID and BIM deficient cells, as well as BAX and BAK double-knock-out cells, are protected from cell death by external ER insults (Ren et al., 2010; Almanza et al., 2018). The extrinsic apoptotic pathway could also be upregulated under ER stress conditions. Thus, CHOP and ATF4 have been shown to increase the expression of DR4 and DR5 receptors (Hiramatsu et al., 2015; Iurlaro et al., 2017). In fact, bortezomib have been shown to cooperate and potentiate cell death induced by Apo2L/TRAIL in MM cell lines (Balsas et al., 2009).

Bcl-2 family are better known for their roles in controlling mitochondrial permeability and cell death mechanisms. However, they also play important roles in regulating calcium ER homeostasis and ER stress-induced cell death. Interestingly, an intense crosstalk between mitochondria and ER organelles exists, which even increases during ER stress conditions (Bhat et al., 2017). For example, BAX and BAK are capable of modulating IRE1 activity during ER stress by interacting with IRE1 (Hetz, 2006). In similar way to mitochondria, BAX and BAK can also oligomerize at the ER membrane under ER stress conditions. This results in an increase of ER-membrane permeability and the release of ER resident proteins such as calreticulin, BIP, PDI and GRP94, which could aggravate ER stress and ROS production (Rodriguez et al., 2011; Pihán et al., 2017). This process is triggered by BH3-only members and counteracted by Bcl-2 and Bcl-x_L_ (Wang et al., 2011; Kanekura et al., 2015)_._ The mechanism by which ER permeabilization leads to cell death is still unknown. However, it has been speculated that ER permeabilization could bring about release of ER-Ca^2+^ stores and increase Ca^2+^ flux to the mitochondria through mitochondria ER-associated membranes (MAMs). This would instigate cell death by mitochondrial permeabilization transition pore (mPTP) (Pihán et al., 2017). Taken together, these studies delineate the ER as an important stress sensor and integrator where also cell fate decisions may take place, with Bcl-2 family as the critical circuitry that connect and modulate the mechanisms involved in cell fate (UPR, apoptosis and also autophagy).

## Immunotherapy in MM

**FIGURE 3:**
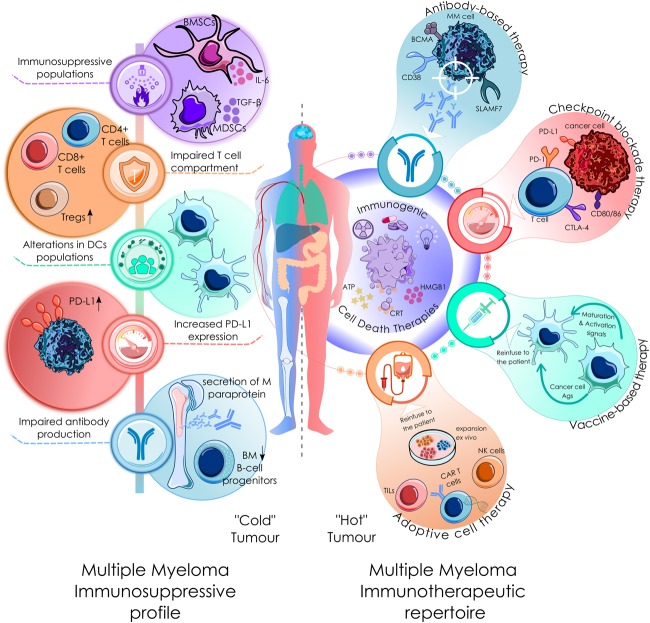
Current clinical immunological scenario in MM. MM is a genuine example where the immune system is compromised. It is characterized by: (1) Deficits in antibody production due to a reduction of bone marrow (BM) B-cell progenitors (Rawstron et al., 1998). (2) General disruption of T-cell immune profile, characterized by increased numbers of regulatory T cells (Tregs), aberrant CD4/CD8 ratios and altered CD4+ T cell numbers among others (Braga et al., 2014; Joshua et al., 2016; Chen et al., 2017). (3) Increased expression of programmed cell death ligand 1 (PD-L1), one of the immune checkpoint inhibitory ligands that counterbalance T cell activity (Paiva et al., 2015; Jung et al., 2017). (4) MDSCs and BMSCs are also a major issue in MM. They are important players sculpting a permissive BM microenvironment, through cell-to-cell (Mondello et al., 2017) o exosome-mediated contacts (Wang et al., 2014) with MM cells, they secrete cytokines that favor the recruitment of immunosupressive populations such as Tregs. (5) An impaired DC function and alterations in DCs frequencies and phenotypes have been found in MM patients (Pasiarski et al., 2013; Leone et al., 2015; Brown et al., 2018). At the right is depicted the current immunotherapeutic repertoire in MM therapy. All these immunotherapeutic approaches could be virtually benefited from the immunostiumulatory effect of ICD-related therapies.

Multiple myeloma is a hematological malignancy that arises due to uncontrolled proliferation of abnormal plasma cells. It accounts for 10–20% of all hematological neoplasms and 0.9% of all newly diagnosed cancer cases worldwide (Bray et al., 2018). Over the past two decades, treatment regimens and survival rates of myeloma patients have witnessed a radical improvement, with ASCT, IMiDs, proteasome inhibitors and monoclonal antibodies as the contributors to this advance. Among them, proteasome inhibitors, stand out as the cornerstone of this scientific and medical achievement (Scalzulli et al., 2018). However, although overall survival and patient outcomes have considerably improved, drug resistance is still a major concern and accounts for the fatality of the disease (Nikesitch et al., 2018; Robak et al., 2018). That is why novel and more efficient (immuno)therapeutic approaches may take the relief. It is important to point out that MM is a genuine example where the immune system is compromised. Deficits in antibody production/immunoglobulin levels due to a reduction of bone marrow (BM) B-cell progenitors are common in MM (Rawstron et al., 1998). General disruption of T-cell immune profile has also been observed, characterized by increased numbers of regulatory T cells (Tregs), aberrant CD4/CD8 ratios and altered CD4+ T cell numbers among others (Braga et al., 2014; Joshua et al., 2016; Chen et al., 2017). MM is also characterized by augmented expression of programmed cell death ligand 1 (PD-L1), one of the immune checkpoint inhibitory ligands that counterbalance T cell activity by binding to PD-1 on activated T cells (Paiva et al., 2015; Jung et al., 2017). MDSCs are also a major issue in MM, as expansion of this population usually correlates with disease progression and a negative clinical outcome (Malek et al., 2016). In addition, MM also finds good allies in BM stromal cells (BMSCs), which are important players sculpting a permissive BM microenvironment (Mahindra et al., 2010). Through cell-to-cell (Mondello et al., 2017) o exosome-mediated contacts (Wang et al., 2014) with MM cells, they secrete cytokines that favor the recruitment of immunosupressive populations such as Tregs and MDSCs (Giallongo et al., 2016; Malek et al., 2016). Finally, several studies have documented an impaired DC function and although contradictory results have been reported, alterations in DCs frequencies and phenotypes have been found in in MM patients (Pasiarski et al., 2013; Leone et al., 2015; Brown et al., 2018). Despite all these stones in the immunotherapeutic path, immune-interventions have potential to be successful in this disease. Graft-vs-myeloma effect was firstly evidenced in patients subjected to ASCT or under donor lymphocyte infusions, suggesting an active immune response against myelomatous cells (Ladetto et al., 2016). Current immunotherapeutic approaches that are giving positive results in relapsed and refractory patients are going to be described below (see also Figure 3).

### Antibody-Based Therapy

Although monoclonal antibodies (moAbs) have been in the anticancer therapeutic armamentarium for some years, effectively treating some solid and hematological cancers, it was only a few years ago that Daratumumab was approved for the treatment of MM. Daratumumab is a moAb that selectively targets CD38, an antigen highly expressed in aberrant plasma cells and at relatively low levels on normal lymphoid and myeloid cells, including normal PCs. Similarly, other anti-CD38 moAbs are currently under investigation such as isatuximab and MOR22. As single agent, Daratumumab showed a promising efficacy, observing objective response rates (ORRs) of approximately 30%, progression free survival (PFS) of 4 months and overall survival (OS) of 20 months, in relapsed and refractory MM (RRMM) patients heavily treated with at least two prior lines of therapy (Lokhorst et al., 2015; Lonial et al., 2016; Rodríguez-Otero et al., 2017). Daratumumab has been shown to kill MM cells through a plethora of mechanisms ranging from antibody-dependent cell mediated cytotoxicity (ADCC) mediated by NK cells, complement-medicated cytotoxicity (CDC), antibody-dependent cell phagocytosis (ADCP) mediated by macrophages and even apoptosis via direct cross-linking (van de Donk and Usmani, 2018). NK cell-mediated cytotoxicity seems to be one of the main mechanisms, and since patient NK cell status may vary, this could explain differences in response between patients (van der Veer et al., 2011). Nowadays, another moAb, elotuzumab, has been approved in MM therapy targeting the SLAMF7 molecule expressed among normal and myeloma PCs, NK, and T cells. The mechanism of action of Elotuzumab is thought to differ from that of daratumumab. This thought is based on the fact that elotuzumab alone has not reached objective responses in MM patients but when combined with lenalidomide and dexamethasone, in a phase II trial and afterward in the Eloquent-2 phase III trial, significantly improved ORRs and OS in RRMM patients (Lonial et al., 2015; Rodríguez-Otero et al., 2017).

Combination of chemotherapy with this kind of approach could render synergistic effects and improve patient’s outcomes. Interestingly, IMiDs have shown to prime MM cell lines to Daratumumab-induced NK cell-mediated cell death (Fedele et al., 2018). In fact, several clinical trials combining IMiDs and Daratumumab have been performed obtaining good results (Gavriatopoulou et al., 2018). Similarly, the efficacy of Daratumumab alone was even improved with combination regimens of daratumumab plus lenalidomide and dexamethasone or daratumumab with bortezomib plus dexamethasone, significantly extending PFS period with strong and durable responses (Blair, 2017; Rodríguez-Otero et al., 2017). As Carfilzomib has shown better survival curves compared to bortezomib, combinations of Daratumumab plus carfilzomib and dexamethasone are currently under phase I investigation (clinical trial NCT03158688).

Other novel and promising designs of these kind of therapy are the conjugated antibodies and the bi-specific T cell engagers (BiTEs). Conjugated antibodies carry in their structure cytotoxic molecules that are guided by the specificity of the antibody part and delivered directly into the target. In particular, an anti-BCMA specific antibody linked to a new class of antimitotic agent, monomethyl auristatin F, has been developed (GSK2857916). This formulation has demonstrated in a phase I trial a 60% response rate and PFS of 7.9 months in RRMM patients with at least three prior lines of therapy (Trudel et al., 2018). Regarding the BiTEs, these are bispecific antibodies that hold on one side specificity for the target cancer cell epitope and on the other recognizes (generally) CD3 molecules on T cells facilitating the contact between them. This way, contact between effector cells and cancer cells is facilitated. There are several BiTEs targeting the BCMA antigen that are currently under development (BI 836909, EM801 and JNJ-64007957) and showed positive results in preclinical models (Cho et al., 2018). Some of these have now entered clinical trials (NCT02514239, NCT03145181, NCT03269136 and NCT03269136), we will have to wait to new updates of these and other studies to check the efficacy of these new formulations.

### Adoptive Cell Therapy

Another way to confront the tumor is by directly using and improving patient’s own defenses (immune effector cells) to kill cancer cells with ACT. By expanding, activating and even engineering NK or T cells outside the immunosuppressive tumor microenvironment, some of the immune barriers may be successfully, or at least partially overcome. As mentioned earlier, graft versus myeloma effect has been observed in patients subjected to autologous stem cell transplantation (ASCT). This effect is thought to be mainly mediated by T cells. Therefore, this population and more specifically, tumor infiltrating lymphocytes (TILs), MILs in the case of myeloma, represents one of the major immune effector cells that could be used to fight MM. Although clinical data in this issue is still scarce, encouraging results has been reported. Noonan et al. (2015) reported that a 90% reduction of tumor burden was achieved with a PFS of 25.1 months, hence demonstrating the feasibility and efficacy of this approach. Genetically engineered T cells stand as a novel and a leading therapeutic opportunity in cancer in general and also in MM. There are two categories: (1) Transgenic TCRs, with specificity toward a tumor antigen in the context of MHC molecule and (2) chimeric antigen receptor (CAR) T cells, which are fusion proteins composed of a single-chain variable fragment (scFv) that directs the specificity toward the cancer cell antigen, coupled to intracellular signaling modules (CD3ζ) or costimulatory molecules (CD28 or CD137/4-1BB). TCR engineered T cells have the advantage to recognize both intracellular and surface antigens, therefore virtually any tumor antigen could be targeted. However, they are restricted to the HLA-I type limiting the patient eligibility criteria (Rodríguez-Otero et al., 2017). Moreover, potential recombination with TCR α and β chains could lead to off-target toxicities due to generation of unexpected MHC-TCR-peptide complex (Cohen, 2018). Fatal and sudden toxicities have been observed in two patients receiving transgenic TCR T cells with specificity to MAGE-A3 class I peptide, due to unwanted specificity of transgenic TCR toward the myocardial protein titin (Linette et al., 2013). Therefore, caution in selecting the proper Ag must be taken. In myeloma, transgenic TCR T cells for NY-ESO1 peptide and its homolog LAGE are currently under clinical testing (Rapoport et al., 2015). Regarding the use of CAR T cells, one of its limitations is that only surface antigens can be targeted, so the number of available targets is lower with this approach. Therefore, the success of this therapy relies on selecting the appropriate target, to selectively kill the cancer cell limiting off-target and targeted-toxicities on healthy tissue. To date CD19 CAR T cells has shown remarkable results on acute lymphoblastic leukemia, chronic lymphocytic leukemia and non-Hodgkin lymphoma (Porter et al., 2015; Maude et al., 2018). Nowadays, there are several antigens in the anti-myeloma CAR T cell repertoire including CD19, CD138, CD38 and SLAMF7. To date BCMA CAR T cell formulation is the one that has been developed in further extent (Cohen, 2018). Several clinical trials have tested or are currently testing BCMA CAR T cells in heavily treated RRMM patients reporting encouraging results. In these studies, overall response rates were close to 80% or even higher and CRs were achieved in an important proportion of patients (Castella et al., 2018; Cohen, 2018).

Similarly, NK cells also pose as a committed ally in cancer therapy. They do not rely on MHC restriction or antigen recognition, but rather they are dependent on the balance between activating and inhibitory receptors. In MM, NK cell numbers and functionality are usually altered, therefore it is feasible to think that restoration of NK cell compartment with ACT could represent a suitable opportunity to face this disease. There are many therapeutic options that are currently under clinical evaluation. They mainly differ in their source (umbilical cord vs. peripheral blood), in their allo-reactivity (autologous vs. allogeneic), and the expansion and stimulation protocols used to prepare and improve these cells (Fionda et al., 2018). One conclusion may be drawn out from all these studies and that is the superior capacity of allo-reactive NK cells to bring myeloma down. Regarding the use of CAR NK cells in MM, they are still under preclinical studies and have not move yet to clinical investigation.

Here chemotherapy could also improve the effectiveness of these approaches. In particular, Lenalidomide has shown to improve the function and persistence of anti-myeloma CS1 CAR T cells *in vivo* (Wang et al., 2018). Carfilzomib has also shown activating and sensitizing activities over NK cells and MM cells, respectively (Chang et al., 2018). In addition, the combination of expanded and activated allogeneic NK cells (eNK) with therapeutic mAbs directed against tumor antigens (e.g., daratumumab in the case of MM), could give excellent results through ADCC mediated by eNK cells (Sanchez-Martinez et al., 2018).

### Releasing the Brakes With Checkpoint Blockade

T cell activation is a complex and well-regulated process. When the menace have been removed, returning to the homeostatic state and preventing damage of tissues requires negative feedback signals that terminate with the immune response. To that end, checkpoint inhibitors are the major class of receptors that provide these attenuation signals to limit the T cell response. Multiple inhibitory checkpoints have been discovered so far: CTLA-4, PD-1, LAG-3, TIM-3, etc. Although, currently both stimulatory and inhibitory checkpoints are under investigation, the checkpoint drugs on which clinical therapies have been developed are CTLA-4, PD-1 and PD-L1. CTLA-4 is an inhibitory receptor expressed on activated T cells and binds to B7 costimulatory molecules on APCs with higher affinity than CD28. Therefore, CTLA-4 blocks and displaces costimulatory interactions eventually leading to abrogation of T cell activation. Ipilimumab, a blocking antibody against CTLA-4, was the first of these type of drugs clinically tested, showing important improvements in metastatic melanoma patients (Robert et al., 2011; Sharma and Allison, 2015). Like CTLA-4, PD-1 is also a checkpoint inhibitory receptor expressed on activated T cells and has two known ligands, PD-L1 and PD-L2. PD-1/PD-L1 (PD-L2) signaling axis interferes with TCR signaling and contributes to T cell exhaustion. PD-L1 / PD-L2 are widely expressed among different cell types and their expression is known to increase under IFN-γ exposure (Sharma and Allison, 2015). Hence, it is thought that this pathway is a late mechanism of protection from T cell activation and represents a physiological way to regulate termination of inflammatory reactions (Sharma and Allison, 2015; Cogdill et al., 2017). PD-L1 is upregulated in tumor cells acting as a disguise mechanism that allow them to escape from T cell-mediated tumor surveillance. Moreover, PD-L1 expression has been linked with poor prognosis in a variety of human cancers (Ghebeh et al., 2006; Mu et al., 2011). On the other hand, probably due to the immunosuppressive character of the tumor microenvironment, TILs show higher expression of PD-1 (Fourcade et al., 2010; Zhang et al., 2010). In MM, PD-L1 expression is upregulated on myeloma cells but not in normal plasma cells from healthy donors (Liu et al., 2007; Tamura et al., 2012; Paiva et al., 2015; Yousef et al., 2015). In fact, higher PD-L1 expression in MM cells was associated with disease progression as shown in the differences of PD-L1 expression between MGUS, MM and relapsed/refractory MM (RRMM) patients (Paiva et al., 2015). Blocking PD-1 alone with nivolumab has not reached good clinical objective responses with half of the patients experiencing disease stabilization in a phase I study (Lesokhin et al., 2016; Rodríguez-Otero et al., 2017). Similarly, on KEYNOTE-013 study, Ribrag and colleagues assessed the clinical efficacy of the anti-PD-1 mAb pembrolizumab as single agent in patients with RRMM. No patient of the 30 enrolled in the study experienced any response and the best outcome observed was again disease stabilization (Paul et al., 2018).

Although checkpoint blockade therapy alone has shown promising results in some cancer patients, this response is not universal and strongly relies on the tumor microenvironment. Thus, checkpoint blockade efficacy may also be refined by induction of more propitious immunogenic conditions in the tumor tissue through ICD. Recent preclinical studies have shown that immunogenic chemotherapy may sensitize cancer cells to checkpoint blockade leading to synergistic responses. In a lung mouse cancer model, an approved clinical chemotherapy regimen (Oxaliplatin plus cyclophosphamide) were able to foster CD8^+^ T cell infiltration and increase TLR4^+^ DCs in tumor tissue, which leads to sensitization of tumors to immune checkpoint therapy (Pfirschke et al., 2016). Another study also showed that the CDK inhibitor dinaciclib was able to increase immune infiltration and activation within tumors and combination with anti-PD1 therapy resulted in enhanced anticancer activity in three different syngeneic mouse cancer models (Varpe et al., 2012). In the clinical practice, NSCLC patients treated with combined regimens of chemotherapy (platinum-based) with different anti-PD1 agents have demonstrated considerable higher response rates and improved clinical outcome compared to that seen on single-agent modalities (Mathew et al., 2018). In patients with metastatic renal cell carcinoma, combination of anti-PD1 (nivolumab) plus pazopanib or sunitib also showed promising clinical responses (Amin et al., 2014).

In MM, preclinical data shows that lenalidomide, one of the so-called immunomodulatory drugs (IMiDs), reduce the expression of PD-1 and PD-L1 in MM cells and BM accessory cells isolated from RRMM patients. Moreover, a synergistic effect between lenalidomide and anti-PD-1 or anti-PD-L1 was observed (Görgün et al., 2015). These results encouraged the rationale of using PD-1/PD-L1 blockade in combination with IMiDs in the treatment of MM. Hence, phase I and phase II clinical trials on RRMM patients who underwent at least three prior lines of therapy have been conducted (Wilson et al., 2016; Badros et al., 2017). These studies showed ORRs of 60% with even some cases of complete response. Therefore, development of phase III clinical trials were the following step to test these combination modalities (Malavasi et al., 2018). Pembrolizumab plus Len and Dex (KEYNOTE-185, NTC02579863), Pembrolizumab plus Pom and Dex (KEYNOTE-183, NTC02576977) and another phase III study testing three different combination regimens (Poma and Dex vs. nivolumab, Pom and Dex vs. nivolumab, elotuzumab, Pom and Dex; CheckMate 602, NCT02726581) were developed. However, these studies were discontinued due to an increase of unprecedented deaths in the pembrolizumab group as well as that no objective responses were observed in the tested groups.

### DC-Based Vaccines and Its Enhancement/Upgrade With ICD

Due to its particular nature, DCs are at the fine-tuned crossroads between innate and adaptive immunity, playing a pivotal role in anti-cancer host immune responses. Therefore, DC-based vaccines seem to be a good option to re-educate the host immune system against myeloma, leading not only to the expansion of anti-tumor specific T cells, but also to long-term memory generation. Since its first documented clinical use on melanoma patients in 1995 (Mukherji et al., 1995), DC-based vaccines have gained momentum in anti-cancer therapy. In fact, this approach has showed positive survival benefits in a diverse set of human cancers (Kantoff et al., 2010; Nakai et al., 2010; Anguille et al., 2014; Cao et al., 2014). In the particular case of MM, DC-MM fusion vaccines achieved anti-cancer immune responses and disease stabilization in the vast majority of patients (Rosenblatt et al., 2011; Rosenblatt et al., 2013). In hematological cancers, following ASCT a complete “resetting” of the hematological system occurs, leaving a huge opening to vaccination strategies to succeed (Rodríguez-Otero et al., 2017). However, although considerable objective clinical responses have been observed, the overall clinical outcome still has not reached the expected standards (Anguille et al., 2014; Vandenberk et al., 2015). As mentioned earlier, due to the hostile microenvironment surrounding MM cells, DC populations are dysfunctional in MM, showing impaired T-cell stimulation capacity (Guillerey et al., 2016; Chung, 2017). Moreover, it is said that the antigens displayed by myeloma cells are presented to DCs in absence of the appropriate costimulatory signals. Therefore, these interactions lead to inadequate immune responses and even create tolerance against cancer Ags (Chung, 2017). For these reasons, there is a consensus that DC vaccines may need to be optimized and standardized in order to enhance their clinical efficacy. There are several factors that have a direct impact on DC biology and the quality and potency of the ensuing T cell responses: route of administration and frequency of injection, delivery system, use and type of adjuvants, nature of DC vaccine formulations, and nature of tumor cell lysates/antigen cargo (Vandenberk et al., 2015; Rodríguez-Otero et al., 2017). Among them, the immunogenicity of dying cancer cells used to load DCs could be easily and notably improved by using ICD-inducers. Numerous studies have proven the potential of ICD-inducers to have a huge impact on DC biology and improve the ability of DCs to stimulate effector cells and enhance anti-cancer T cell responses *in vivo*. For example, γ-irradiation, has been shown to effectively induce DCs maturation and stimulate *in vivo* CTL responses (Goldszmid et al., 2003). Moreover, γ-irradiated cells efficiently immunized mice against a subsequent rechallenge with live syngeneic cancer cells in various preclinical models (Strome et al., 2002). Different ICD-related modalities such as UV light (Brusa et al., 2008), oncolytic viruses (Donnelly et al., 2011), HHP (Mikyšková et al., 2016), heat shock (Adkins et al., 2017) among others have shown to upregulate maturation markers in DCs as well as prime antigen specific T-cell responses both *in vitro* and *in vivo*. Hyp-PDT is also equally effective in inducing complete tumor regression *in vivo* both in curative and prophylactic vaccination settings (Sanovic et al., 2011). DCs charged with Hyp-PDT treated cells significantly enhanced CTL responses, IFN-γ producing CD8^+^ T cells and Th1-driven immunity in ectopic murine mammary tumors (Jung et al., 2012) as well as orthotopic glioma mice models (Garg et al., 2016).

In the clinical practice, melanoma and high-grade glioma patients have successfully been treated with DC vaccines loaded with γ-irradiated tumor cells (Cho et al., 2012). In the case of glioblastoma multiforme, patients who underwent conventional treatment plus DC-based therapy showed an increased short-term (1–3 years) survival rates compared to control group receiving conventional therapy (Cho et al., 2012). Relapsed Non-Hodgkin’s B-cell lymphoma (NHL) patients have also benefited from DC vaccines pulsed with γ-irradiated, heat shock or UV light-treated tumor cells (Zappasodi et al., 2010). Accordingly, CRT and HSP90 expression levels on NHL cells positively correlated with the observed clinical and immune responses (Zappasodi et al., 2010).

In MM, data regarding the use of ICD-dying cells to provide an enhanced immunogenic feed to DCs and the expected *in* *vivo* anti-cancer immune responses are still lacking. In particular lenalidomide has shown to impact DCs biology and enhance CD8^+^ T cell cross-priming by primed DCs (Henry et al., 2013). Another study evaluated ICD induced by bortezomib in MM cell lines and MM primary cells, as well as the capacity of bortezomib-treated cells to increase maturation markers in DCs and to induce proliferation and polarization toward IFN-γ producing T cells *in vitro* (Spisek et al., 2007). There is currently an ongoing phase II clinical trial testing DC/MM fusion vaccines in combination with lenalidomide and GM-CSF (NCT02728102). We will need to wait for further studies to see the clinical advantages of combining this type of approaches.

## Concluding Remarks and Future Perspectives

Over the past years, ICD and ER stress are gaining momentum in anti-cancer therapy. The ability of chemotherapeutics and other anti-cancer therapies, not only to mount an active immune response against the tumor, but also to modulate the cancer immune environment, has transformed the therapeutic scenario in oncoimmunology. Moreover, understanding of the molecular pathways involved in all these processes, is uncovering a whole new set of potential prognostic biomarkers with which cancer patients could be better monitored and stratified to determine their optimal therapeutic regimen. However, given that certain danger signaling markers have been found both in treated and untreated patients, further investigations are needed to unravel the real repercussion of therapy driven-ICD, as well as oncogenic-driven DAMP exposure in the clinical setting. Furthermore, special caution is needed when targeting ER stress and UPR pathways, as it could pose both beneficial and detrimental consequences on patient’s outcome. On one sid, we may be enhancing cell death pathways or boosting immunogenicity of cell death, but on the other we could also be fostering the cytoprotective function of the UPR as well as some ER stress-related immunosuppressive effects. Nonetheless, given the adaptability and complexity of cancer, it is becoming increasingly clear that future anti-cancer therapeutic approaches will take advantage from combination of immunogenic (chemo)therapeutic modalities with current and novel immunotherapeutic regimens. In particular, in MM, this type of combinatorial approaches have a great opportunity to success, since encouraging results have been already obtained. Nonetheless further investigations awaits to circumvent and manage some of the basic problems and clinical adverse events that arise with these novel kind of approaches.

## Author Contributions

All authors wrote the review and revised the bibliography. AS performed the figures.

## Conflict of Interest Statement

The authors declare that the research was conducted in the absence of any commercial or financial relationships that could be construed as a potential conflict of interest.
